# The amyloid precursor protein derivative, APP96-110, is efficacious following intravenous administration after traumatic brain injury

**DOI:** 10.1371/journal.pone.0190449

**Published:** 2018-01-10

**Authors:** Stephanie L. Plummer, Frances Corrigan, Emma Thornton, Joshua A. Woenig, Robert Vink, Roberto Cappai, Corinna Van Den Heuvel

**Affiliations:** 1 Translational Neuropathology Laboratory, The University of Adelaide, Adelaide, South Australia, Australia; 2 Division of Health Sciences, The University of South Australia, Adelaide, South Australia, Australia; 3 Department of Pathology, The University of Melbourne, Melbourne, Victoria, Australia; University of Florida, UNITED STATES

## Abstract

Following traumatic brain injury (TBI) neurological damage is ongoing through a complex cascade of primary and secondary injury events in the ensuing minutes, days and weeks. The delayed nature of secondary injury provides a valuable window of opportunity to limit the consequences with a timely treatment. Recently, the amyloid precursor protein (APP) and its derivative APP96-110 have shown encouraging neuroprotective activity following TBI following an intracerebroventricular administration. Nevertheless, its broader clinical utility would be enhanced by an intravenous (IV) administration. This study assessed the efficacy of IV APP96-110, where a dose-response for a single dose of 0.005mg/kg– 0.5mg/kg APP96-110 at either 30 minutes or 5 hours following moderate-severe diffuse impact-acceleration injury was performed. Male Sprague-Dawley rats were assessed daily for 3 or 7 days on the rotarod to examine motor outcome, with a separate cohort of animals utilised for immunohistochemistry analysis 3 days post-TBI to assess axonal injury and neuroinflammation. Animals treated with 0.05mg/kg or 0.5mg/kg APP96-110 after 30 minutes demonstrated significant improvements in motor outcome. This was accompanied by a reduction in axonal injury and neuroinflammation in the corpus callosum at 3 days post-TBI, whereas 0.005mg/kg had no effect. In contrast, treatment with 0.005m/kg or 0.5mg/kg APP96-110 at 5 hours post-TBI demonstrated significant improvements in motor outcome over 3 days, which was accompanied by a reduction in axonal injury in the corpus callosum. This demonstrates that APP96-110 remains efficacious for up to 5 hours post-TBI when administered IV, and supports its development as a novel therapeutic compound following TBI.

## Introduction

Traumatic brain injury (TBI) is a major public health concern, one which in industrialised countries leads to more deaths in people under the age of 45 than any other cause [1]. Following TBI, extensive neurological damage occurs through the initiation of multiple injury events, both immediately after and progressively from the initial injury. This delayed injury is a prolonged and deleterious cascade of cellular and biochemical events that often compound the existing injury including excitotoxicity, oxidative stress, irreversible cell injury and death, inflammation and blood-brain-barrier (BBB) disruption [2–5]. Diffuse axonal injury (DAI), a significant feature of TBI, is the most common cause of coma and vegetative state following head trauma [6, 7]. Occurring as a result of rapid acceleration/deceleration forces, DAI causes considerable deformation of brain tissue through shearing forces and stretching, and currently lacks an efficacious pharmacological treatment. Many of these serious events could be reversed if targeted with an appropriate therapy, preventing serious complications and reducing the burden on society. Fortunately, the delayed onset and potentially reversible nature of these secondary events provides a novel window of opportunity for a therapy to reduce neuronal damage and help limit the associated morbidity and mortality of TBI [3]. It has been proposed that upcoming therapeutic interventions should be multifactorial in nature and target multiple elements of the secondary injury cascade [3, 8, 9]. One frequently proposed approach is to emulate the brain’s endogenous repair response.

It has been consistently demonstrated that the amyloid precursor protein (APP) and its derivatives are neuroprotective following experimental TBI [10–14]. Intracerebroventricular administration of APP was shown to ameliorate both motor and cognitive deficits, as well as attenuate cellular loss and inflammation following TBI *in vivo*. This makes APP a promising candidate to develop as a therapeutic treatment for TBI patients. APP undergoes distinct proteolytic events [15], and the soluble APPα (sAPPα) species, produced via proteolytic cleavage with α-secretase enzyme, is thought to be responsible for APP’s neuroprotective properties following TBI, including protection against a range of cytotoxic insults, regulation of neurite outgrowth and synaptogenesis, and improvement in functional outcome *in vivo* [16–18]. Domain mapping studies attributed the neuroprotective activity of sAPPα to the growth factor domains [11], with subsequent sequence activity studies able to localize the neuroprotective activity to the N-terminal heparin binding region, encompassed by amino acid residues 96–110 [14]. It was shown that APP96-110’s neuroprotective activity correlated with its affinity for heparin [11, 14, 19]. Intracerebroventricular administration of APP96-110 was shown to not only restore motor and cognitive deficits, but also preserve cortical and hippocampal tissue in APP-/- mice, and reduce axonal injury in the corpus callosum following diffuse TBI in rats. This suggests that that APP96-110 could functionally substitute for native sAPPα [14].

Whilst these results using intracerebroventricular administration are promising and clinically relevant, the intravenous (IV) route of administration would provide a more tractable method of drug administration, especially in a paramedic setting where TBI victims will first encounter medical treatment. Accordingly, this study aimed to determine the efficacy of IV administered APP96-110, along with its therapeutic window, following moderate-severe diffuse TBI. APP96-110 was firstly administered IV at 30 minutes after trauma, and its effects on motor outcome, the extent of DAI and neuroinflammation at 3 days were assessed. Secondly, delayed IV administration of APP96-110 at 5 hours post-TBI was assessed to determine if APP96-110 would remain efficacious at a delayed but more clinically relevant time point.

## Methods

All studies were performed within the guidelines established by the National Health and Medical Research Committee of Australia and were approved by the Animal Ethics Committee of the University of Adelaide. All animals were purchased from the University of Adelaide breeding facility.

### The APP96-110 peptide

The APP96-110 peptide, NWCKRGRKQCKTHPH, was synthesized by Auspep (Tullamarine, Victoria, Australia) and was N-terminally acetylated and C-terminally amidated. The peptide was dissolved into distilled water to produce a 1mM stock solution, and further diluted to produce doses of 0.5mg/kg and 0.05mg/kg APP96-110.

### Evaluation of the efficacy of IV APP96-110 administered at 30 minutes following TBI

#### Injury induction

A total of 62 adult male Sprague-Dawley rats weighing between 336g and 427g were group housed in a controlled temperature environment under a 12 hour light/dark cycle, with uninterrupted access to food and water. Animals were randomly assigned into 3 day histological and 7 day outcome groups, and each further assigned into sham, vehicle control, 0.005mg/kg, 0.05mg/kg and 0.5mg/kg APP96-110 treatment groups.

Animals were injured using the impact-acceleration model of diffuse TBI [20]. This injury model produces a rage of injuries from moderate through to moderate–severe, and its heterogeneity provides a clinically relevant model to test neuroprotective therapeutics. Animals were anesthetized with isoflurane and upon the absence of pain reflexes, a midline incision was made on the skull, a metallic disk (10mm in diameter and 3mm in depth) was adhered onto the skull centrally between lambda and bregma sutures. Animals were placed on a foam bed of 10cm height, and injured by releasing a 450g weight from 2 meters onto the metallic disk [20]. The model of TBI is associated with a period of apnea in unventilated animals. To regulate this and replicate what occurs clinically, animals were ventilated directly after injury whilst introducing a period of post-traumatic hypoxia with 2L/min of nitrogen and 0.2L/min of oxygen [21]. Post-traumatic hypoxia has been previously shown to exacerbate both axonal injury and inflammation, and can often be attributed to poor neurological outcome post-TBI [21]. Sham animals were surgically prepared but not injured and did not receive hypoxia. The use of general anaesthetics and the absence of pain receptors in the brain means injury to the brain tissue is not painful for the animals. However, the initial skin incision on the scalp required to adhere the metallic disk can cause minor discomfort in the initial 24 hours post-surgery. As such, all animals received 0.2mL subcutaneous Lignocaine under the incision site at the end of the procedure to minimize pain and discomfort.

At 30 minutes following TBI, animals were administered either saline, 0.005mg/kg, 0.05mg/kg or 0.5mg/kg APP96-110 intravenously via the tail vein by a blinded observer, and returned to their home cage to recover once normal behaviour was established. Intensive continuous monitoring of the animals was provided for the first 2 hours post-procedure and animals were then checked at least hourly for the next 4 hours. If signs of clinical deterioration were evident, the frequency of monitoring was adjusted accordingly so that appropriate intervention could occur. Following this initial period, animals were monitored twice daily with clinical record sheets maintained to assess the following criteria: daily weight loss, appearance and texture of their coats, posture, changes in social behaviour, as well as the presence or absence of stress marks, circling behaviour, OP site swelling, and paresis. Weight loss of greater than 5–10% of their pre-surgical body weight were treated with subcutaneous saline to prevent dehydration. Overall, 9 animals died shortly following the trauma, with a further 9 animals euthanized prior to the end of experiments in accordance with ethical guidelines.

#### Assessment of motor outcome

Motor deficits post-TBI were assessed using the rotarod, a sensitive test of motor ability following TBI [22]. Briefly, the rotarod comprises of 18 rotating metallic rods on which the animal is required to balance and walk. The speed of the rotarod is increased from 6 to 36 rpm in intervals of by 3 rpm every 10 seconds. Animals were pre-trained daily on the rotarod for 5 days until they could complete 120 seconds, and were then assessed daily for 7 days post-injury. The length of time in seconds that animals were successfully able to walk on the rotarod post-injury was recorded.

#### Histological assessment

At 3 days post-TBI, animals were anaesthetized with isoflurane, and upon the absence of pain reflexes, were transcardially perfused with 10% formalin. Animals were perfused at 3 days rather than 7, as the most significant improvements in motor outcome were observed within the first 3 days following trauma. Fixed brains were sectioned into 2mm coronal slices and embedded in paraffin wax, before the brain region containing the area under the impact (-0.40mm relative to Bregma) was further sectioned into 5μm coronal slices for representative serial sections. Immunohistochemistry was performed following standard procedure with sections stained for APP for axonal injury (22C11; Boéhringer, 1:1000), glial fibrillary acidic protein (GFAP; Dako, 1:40,000) and Iba1 (Wako, 1:10,000). Following overnight incubation with the primary antibody, slides were then incubated with the appropriate secondary antibody (1:250, Vector), before incubation with streptavidin peroxidase conjugate (SPC—1:1000). This was followed by antigen detection with 3,3′-Diaminobenzidine tetrahydrochloride (DAB) for 7 minutes, and sections counterstained with haematoxylin. Sections were digitally scanned using the Nanozoomer slide scanner (*Hamamatsu*, Hamamatusu City, Shizuoka, Japan), and the associated software (NDPview) used to view and analyze the images. The extent of DAI was assessed by counting the number of APP immunopositive (APP+) lengths in the entire length of the corpus callosum (from left to right) of two consecutive brain sections per animal, where each animal represents a 10μm area of the corpus callosum. This area is directly under the impact site (-0.40mm relative to Bregma), and was chosen as this region has previously demonstrated the highest degree of axonal injury.

**Fig 1:**
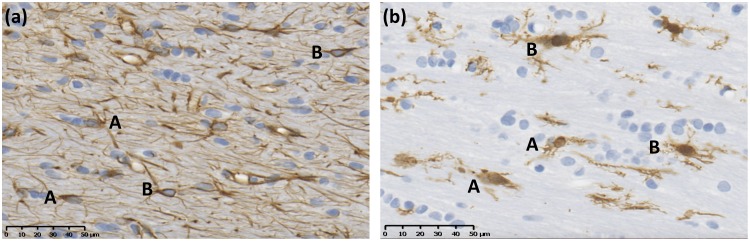
Representative images showing the morphological differences between astrocytes (a) and microglia (b) in the corpus callosum. Astrocytes (a) are characterized morphologically into non-reactive (A) and reactive (B) astrocytes, while microglia (b) are characterized morphologically into inactivated (A) and activated (B) microglia.

GFAP and Iba1 immunoreactivity was determined by assessing morphology (Fig 1). Reactive astrocytes differ from other astrocytes through the increased GFAP expression surrounding the cell body, giving the appearance of a thick and dark halo-like appearance. Furthermore, their processes become shorter, thicker and darker than those of their unreactive counterparts. Conversely, activated microglia can exhibit a number of different morphological characteristics, including changes to the size of the cell body and processes. Microglia demonstrating an increase in length, number and thickness of their processes were of most importance for this study. The number of reactive astrocytes and activated microglia were counted within the same region of the corpus callosum of two consecutive brains sections per animal, as with APP histology. All histological counts were done blinded by two assessors using strict criteria, and minimal inter-observer variation was found.

### Evaluation of the efficacy of IV APP96-110 administered at 5 hours following TBI

A total of 40 adult male Sprague-Dawley rats weighing between 361g and 427g were randomly assigned into 3 day sham, vehicle control, 0.05mg/kg and 0.5mg/kg APP96-110 treatment groups. The APP96-110 peptide was manufactured as described above, and animals were again injured using the impact-acceleration model of diffuse TBI as described above [20]. 4 animals died shortly following the trauma, with the remaining animals monitored appropriately in accordance with ethical guidelines as previously described. Sham animals were surgically prepared but not injured. At 5 hours following TBI, animals were administered either saline, 0.05mg/kg or 0.5mg/kg APP96-110 intravenously via the tail vein by a blinded observer, before being returned to their home cage to recover once normal behaviour was established. Motor outcome and histological assessments were carried out as previously described.

#### Statistical analysis

All data were analyzed using Graph Pad Prism software. All values are displayed as Mean ± SEM, with a significance level of p<0.05. Motor outcome was assessed using a two-way repeated measures ANOVA with Tukey’s post-test. DAI and the number of reactive astrocytes and activated microglia were by assessed using a one-way ANOVA with Tukey’s post-test.

## Results

### Examining the efficacy of IV APP96-110 administered at 30 minutes following TBI

#### Administration of APP96-110 was efficacious on motor outcome

**Fig 2:**
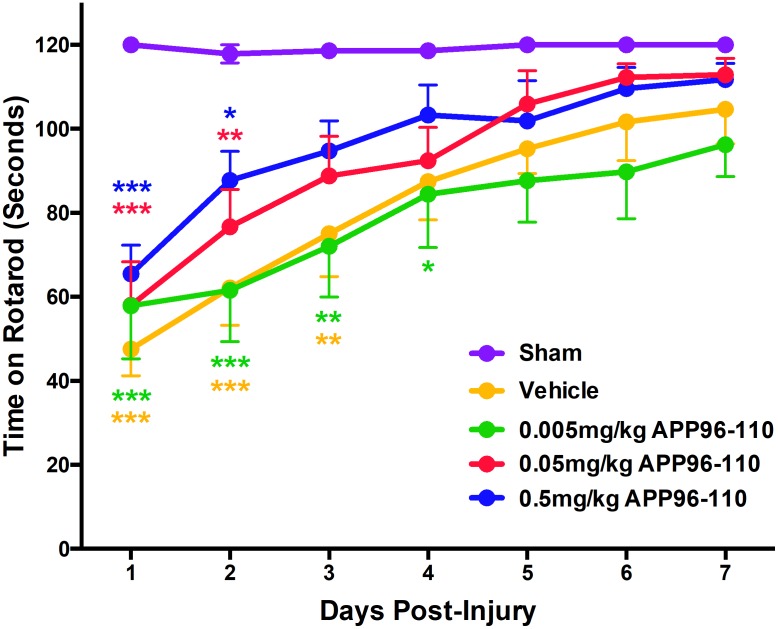
Motor outcome illustrating the effect of treatment with 0.005mg/kg, 0.05mg/kg and 0.5mg/kg APP96-110 IV at 30 minutes post-TBI. Data were assessed with a two-way repeated measures ANOVA followed by Tukey’s post-test. (Sham n = 7, vehicle n = 9, 0.005mg/kg n = 9, 0.05mg/kg n = 9, 0.05mg/kg n = 7, per group). (***p<0.001, **p<0.01, **p<0.05, compared to sham animals).

Motor outcome post-TBI was assessed using the rotarod (Fig 2). Sham animals (n = 7) demonstrated normal motor abilities over the testing period, recording 120 seconds on all 7 days post-injury. In contrast, vehicle control animals showed significant motor deficits when compared to shams on days 1 to 3 post-TBI (p<0.01, n = 9). Animals treated with 0.005mg/kg APP96-110 (n = 9) performed similarly to vehicles, and were significantly different to shams on days 1 to 4 post-injury (p<0.05). However, treatment with both 0.05mg/kg (n = 9) and 0.5mg/kg (n = 7) APP96-110 improved motor outcome over 7 days, as these animals only recorded significant motor deficits different to sham animals on days 1 and 2 post-injury (p<0.05).

#### APP96-110 administration after TBI reduced axonal injury

The finding that only the 0.05mg/kg and 0.5mg/kg doses of APP96-110 were able to significantly improve motor outcome, led us to analyze these doses in histological studies for axonal injury and neuroinflammation at 3 days post-TBI.

**Fig 3:**
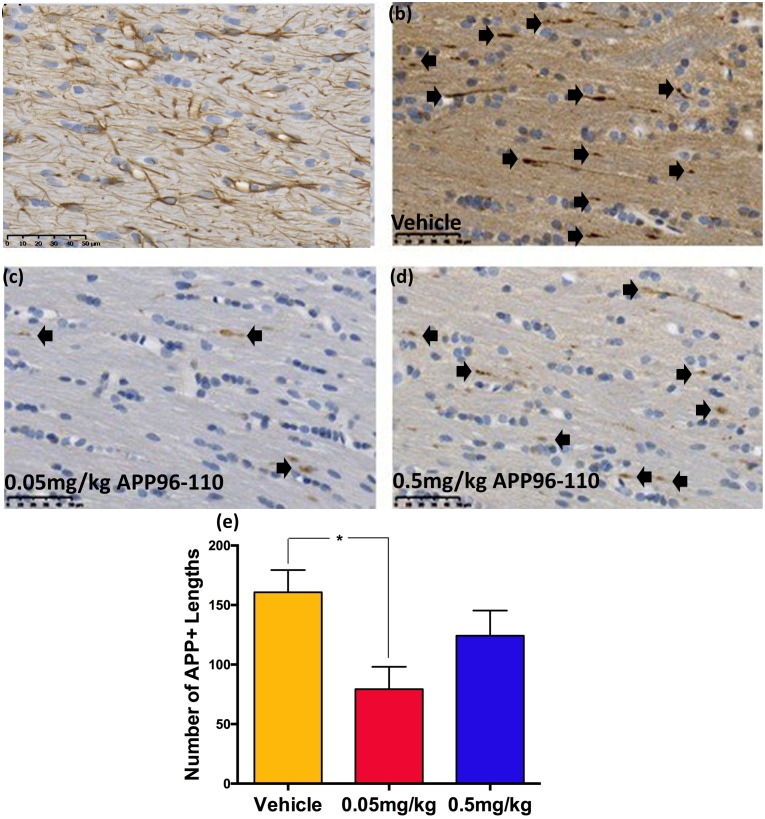
Representative images showing the degree of DAI in the corpus callosum at 3 days following diffuse TBI (a-d). 0.05mg/kg (c) and 0.5mg/kg (d) APP960110 treated animals demonstrated a reduced number of APP immunopositive lengths in the corpus callosum at 3 days following injury, compared to vehicle treated animals (b), with 0.05mg/kg APP96-110 treated animals showing the most pronounced reduction. These observations were confirmed with counts of the number of APP immunopositive lengths (e). APP+ lengths were assessed using a one-way ANOVA followed by Tukey’s post-test. (Sham n = 5, vehicle n = 4, 0.05mg/kg n = 5, 0.5mg/kg n = 4) (*p<0.05, compared to vehicle control animals).

The extent of DAI was determined by counting the number of APP immunopositive lengths within the corpus callosum at 3 days post-TBI (Fig 3). APP is a robust marker for assessing axonal injury [23]. A significant increase in APP immunoreactivity was observed in vehicle control animals at 3 days post-TBI (161±19, p<0.001, n = 4)) when compared to sham animals (p<0.001, n = 5). Administration of both doses of APP96-110 showed a reduction in DAI post-TBI, however, a significant reduction compared to vehicle animals was only recorded in the 0.05mg/kg dose (79±19, p<0.05, n = 5), and not the 0.5mg/kg dose (124±21, n = 4).

#### APP96-110 administration after TBI reduced neuroinflammation

**Fig 4:**
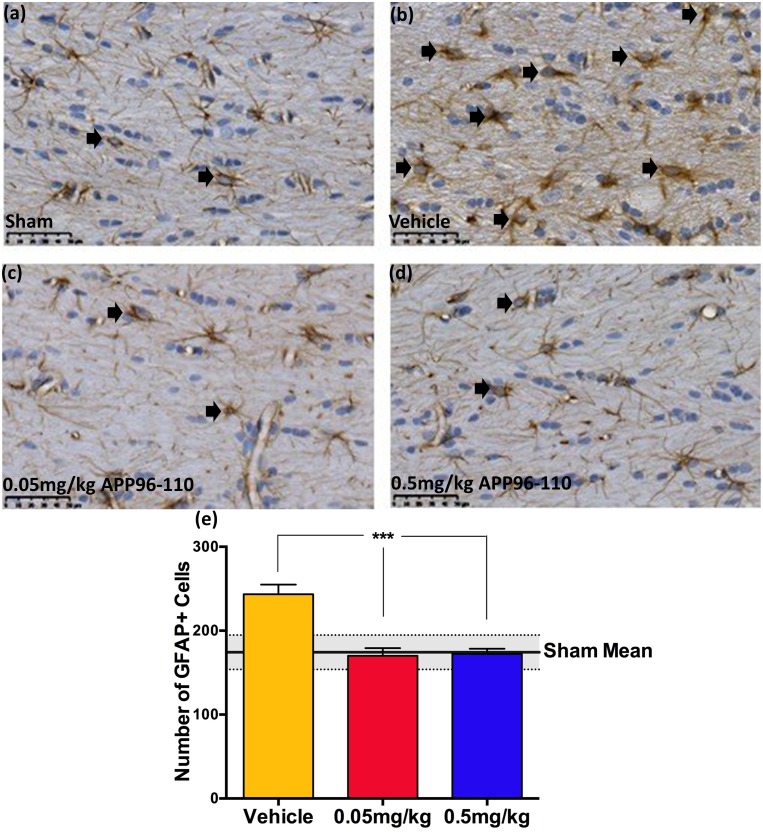
Representative images showing the degree of neuroinflammation in the corpus callosum at 3 days following diffuse TBI, as assessed by GFAP immunoreactivity (a-d). 0.05mg/kg (c) and 0.5mg/kg (d) APP96-110 treated animals demonstrated a clear reduction in the number of reactive astrocytes in the corpus callosum at 3 days following injury, compared to vehicle treated animals (b). These observations were confirmed with counts of the number of reactive astrocytes (e). Positive cell counts were assessed using a one-way ANOVA followed by Tukey’s post-test. (Sham n = 4, vehicle n = 5, 0.05mg/kg n = 5, 0.5mg/kg n = 4) (***p<0.001, compared to vehicle control animals).

**Fig 5:**
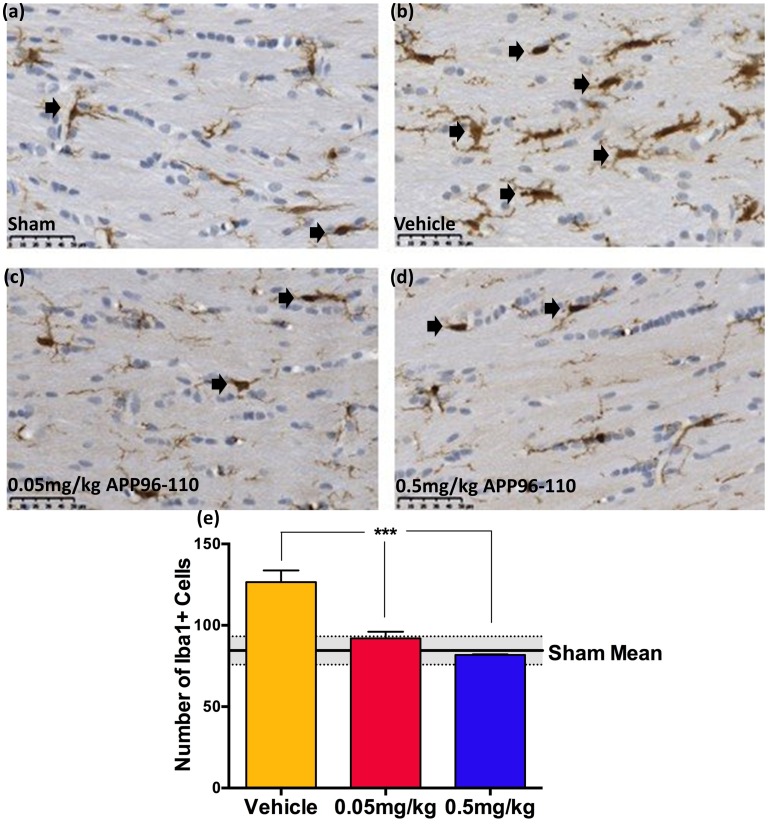
Representative images showing the degree of neuroinflammation in the corpus callosum at 3 days following diffuse TBI, as assessed by Iba1 immunoreactivity (a-d). 0.05mg/kg (c) and 0.5mg/kg (d) APP96-110 treated animals demonstrated a profound reduction in the number of activated microglia in the corpus callosum at 3 days following injury, compared to vehicle treated animals (b). These observations were confirmed with counts of the number of activated microglia (e). The number of positive Iba1 cells was assessed individually using a one-way ANOVA followed by Tukey’s post-test. (Sham n = 4, vehicle n = 5, 0.05mg/kg n = 5, 0.5mg/kg n = 4) (***p<0.001, compared to vehicle control animals).

Neuroinflammation was assessed by counting the number of reactive astrocytes and activated microglia within the corpus callosum at 3 days post-TBI (Figs 4 & 5). GFAP immunoreactivity (Fig 4) demonstrated a significant increase in the number of reactive astrocytes within the corpus callosum in vehicle control animals (243±11, n = 5) at 3 days post-TBI when compared to sham animals (174±10, p<0.01, n = 4). Treatment with both 0.05mg/kg (n = 5) and 0.5mg/kg (n = 4) doses of APP96-110 demonstrated significant reductions in GFAP immunoreactivity (170±9 and 172±6, respectively), compared to vehicle control animals (p<0.001), with levels similar to that seen in shams.

These astrocytic changes were mirrored by Iba1 immunoreactivity (Fig 5) where a significant increase in the number of microglia was observed in the corpus callosum of vehicle control animals (127±7, n = 5) at 3 days post-TBI when compared to sham animals (85±4, p<0.01, n = 4). Notably, animals treated with both the 0.05mg/kg (n = 5) and 0.5mg/kg (n = 4) doses of APP96-110 also demonstrated a return to sham level, with significant reductions in the number of activated microglia in both treatment groups, 92±4 and 82±1, respectively (p<0.001), when compared to vehicle control animals.

### Evaluation of the efficacy of IV APP96-110 administered at 5 hours following TBI

In order to determine whether IV APP96-110 would remain efficacious if delivered at a later and more clinically relevant time point, injured rats were injected 5 hours post-TBI. Rats were treated with either 0.05mg/kg or 0.5mg/kg APP96-110, as these doses were efficacious as determined above.

#### Delayed administration of APP96-110 remained efficacious on motor outcome

**Fig 6:**
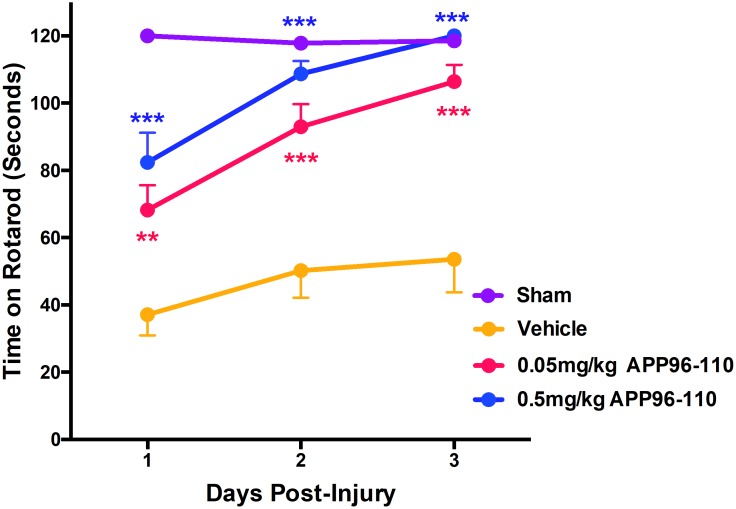
Motor outcome illustrating the effect of treatment with 0.05mg/kg and 0.5mg/kg APP96-110 IV at 5 hours post-TBI. Data was assessed with a two-way repeated measures ANOVA followed by Tukey’s post-test. (Sham n = 7, vehicle n = 9, 0.05mg/kg n = 10, 0.5mg/kg n = 10, per group). (***p<0.001, **p<0.01, compared to vehicle control animals).

As motor deficits were most pronounced on days 1 to 3 post-injury (Fig 2), motor outcome was assessed on these days (Fig 6). Sham animals (n = 7) demonstrated normal motor abilities over the 3 day testing period, recording 120 seconds on all 3 days post-injury, whereas vehicle control animals demonstrated significant motor deficits when compared to shams on all 3 assessment days (p<0.001, n = 9). In contrast, treatment with both doses of APPP96-110 significantly improved motor ability. Animals treated with 0.05mg/kg APP96-110 (p<0.01, n = 10) or 0.5mg/kg APP96-110 (p<0.001, n = 10) demonstrated significant improvements in motor outcome compared to vehicle animals on all 3 days following TBI. (p<0.01). There were no significant differences between the two APP96-110 doses.

#### Delayed administration of APP96-110 remained efficacious in reducing diffuse axonal injury

**Fig 7:**
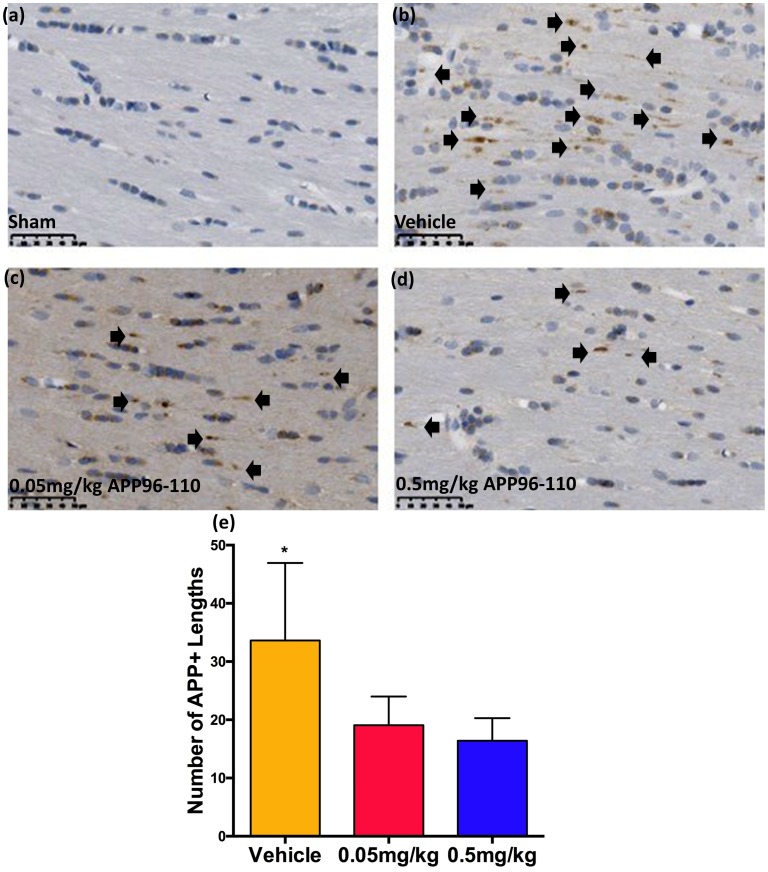
Representative images showing the degree of axonal injury in the corpus callosum at 3 days following diffuse TBI (a-d). 0.05mg/kg (c) and 0.5mg/kg (d) APP960110 treated animals demonstrated a reduced number of APP immunopositive lengths in the corpus callosum at 3 days following injury, compared to vehicle treated animals (b). These observations were confirmed with counts of the number of APP immunopositive lengths (e). APP+ lengths were assessed using a one-way ANOVA followed by Tukey’s post-test. (Sham n = 7, vehicle n = 8, 0.05mg/kg n = 9, 0.5mg/kg n = 9) (*p<0.05, compared to sham animals).

The extent of DAI was determined by counting the number of APP immunopositive lengths in the corpus callosum (Fig 7). Similar to the 30 minute treatment study, there was a significant increase in APP immunoreactivity was observed in vehicle control animals at 3 days post-TBI (34±11, n = 8) compared to sham animals (p<0.05, n = 7). This delayed treatment with APP96-110 remained efficacious and reduced levels in both the 0.05mg/kg (19±11, n = 9) and 0.5mg/kg (16±10, n = 9) treatment groups, with DAI not significantly different to sham animals.

## Discussion

Over recent years, a growing body of data has suggested that APP and its derivatives have a neuroprotective role following TBI [10–12, 24]. This study is the first to examine the neuroprotective efficacy of APP96-110 following IV administration after TBI.

Posttraumatic administration of APP96-110 IV not only demonstrated noticeable improvements in motor outcome and reductions in axonal injury and neuroinflammation, but importantly, that APP96-110 remains efficacious when injected up to 5 hours post-TBI. Previous research has focused solely on intracerebroventricular administration, however, IV administration soon after trauma offers clear clinical advantages. Following trauma, ensuing secondary damage to the BBB facilitates a localized increase in permeability of blood contents into the brain parenchyma as early as 15 minutes after injury, lasting for up to four to six hours for large molecules. Permeability for smaller molecules, however, can last up to three to four days [25, 26]. Whilst this permeability does contribute to the injury process, it also provides a window of opportunity through which therapeutics may gain entry to the brain [26]. A challenge for many IV drugs targeting brain injury is the inability to penetrate the BBB and reach the CNS. However, the ability of IV administered APP96-110 to produce neuroprotective effects *in vivo* that were comparable to intracerebroventricular administration, suggests that APP96-110 can enter the brain following injury in a functional state via the IV route. This has been confirmed with live animal luminescence studies, where APP96-110’s ability to enter the brain was observed for up to 5 hours post-TBI (unpublished data).

Over the years, a number of experimental therapies have shown promising neuroprotective efficacy in experimental settings, but subsequently fail to produce similar neuroprotective efficacy in humans [9, 27]. A therapeutic challenge often slowing bench to bedside progress for TBI is the choice of therapeutic administration time. Research often focuses on an immediate time point after injury, generally up to one hour post-TBI [3, 8]. However, in a clinical setting, the time frame between injury and arrival to a trauma unit can often far exceed one hour [3]. Therefore, testing of more clinically relevant time frames is needed to develop TBI treatments which can be administered by paramedics at the place of injury. APP96-110 demonstrated clear efficacy for up to 5 hours post-injury, a finding that significantly extends the therapeutic timeframe for treatment. Its therapeutic efficacy may in fact extend beyond the 5 hour window, and as such may form the basis of future dose and time response studies using APP96-110.

The results of this study strengthen previous findings that highlight the considerable neuroprotective functions of APP and its derivatives in TBI [18]. Following trauma, IV administration of APP96-110 at 30 minutes resulted in significant improvements in motor outcome over 7 days. Whilst the lowest 0.005mg/kg dose was ineffective, animals treated with the higher doses no longer displayed significant motor deficits compared to shams at two days after TBI. Moreover, these doses were able to reduce the currently untreatable DAI, with the 0.05mg/kg dose of APP96-110 producing a significant decrease in DAI compared to vehicle control animals at 3 days post-TBI. Importantly, when APP96-110 was administered at 5 hours following injury, both the 0.05mg/kg and 0.5mg/kg doses remained efficacious at improving motor outcome, with animals significantly different to vehicle control rats throughout the testing period. This improvement in outcome was also associated with a reduction in levels of DAI in the corpus callosum at day 3 post-injury compared to vehicle control animals. DAI occurs as a result of a progressive secondary insult to axons, often leading to prolonged neurological damage [28–30]. Primary injury often causes considerable axonal stretching, causing localized damage to the cytoskeleton and subsequent disruption to axoplasmic transport. However, much of the progression of DAI is secondary in nature, with the disruption of axoplasmic transport leading to axonal swelling and subsequent axonal disconnection [29, 31], the disruption of sodium and calcium channels facilitating an influx of ions, and the production of deleterious phospholipases and proteases causing damage to mitochondria and complete axonal separation and eventually cell death [7, 31]. Injury to this extent is likely to be a major cause of functional deficits, and a key predictor of functional outcome following trauma, particularly in human CNS conditions.[32] The neuroprotective effects of APP96-110 on DAI, even when administered at the delayed time of 5 hours post-TBI is important, as this suggests that APP96-110 may reduce overall injury severity by limiting the progression of axonal damage throughout the secondary injury cascade, and important finding given that current therapies targeting DAI are lacking.

Whilst axonal injury is an important indicator of injury severity following TBI [7], it is not the sole factor. A hallmark feature of TBI is neuroinflammation, a significant part of the secondary injury cascade, which often leads to the prolonged and detrimental neurological injury and degeneration [33]. As important cells in the neuroinflammatory response, microglia and astrocytes play a key role in a number of both beneficial and detrimental functions following CNS injury, including the release of neurotrophic factors, but also both pro- and anti-inflammatory cytokines [33]. The impact-acceleration model of diffuse TBI produces wide-spread axonal damage throughout the brain, particularly in white matter tracts, like the corpus callosum, where axons are abundant [20]. Whilst neuroinflammation can often be observed in cortical regions, in this setting it was assessed within the corpus callosum, as this region corresponds to the largest area of damage seen following diffuse trauma. Administration of APP96-110 at 30 minutes post-TBI prevented the profound neuroinflammation associated with TBI at all doses tested, with numbers of both reactive astrocytes and activated microglia significantly reduced following APP96-110 treatment. These findings could in part be explained by the associated reductions in DAI seen in APP96-110 treated animals, where the reduction in secondary injury events may reduce the need for neuroinflammation to occur. This is in contrast to vehicle control animals where secondary injury and DAI was more considerable. Furthermore, the reduction in acute neuroinflammation could represent a pathway in which APP96-110 treatment acts to ameliorate the motor deficits. As animals were assessed at day 3, these results only represent the acute neuroinflammatory response, and as such are not reflective of the long-term progressive nature of posttraumatic neuroinflammation. As such, investigation into the mechanism through which APP96-110 is able to reduce neuroinflammatory events remains an important avenue for further research.

While the use of APP96-110 as a novel and clinically relevant therapeutic agent for TBI has been clearly demonstrated, the mechanism of action through which APP96-110 exerts these effects is yet to be fully understood. A key functional domain of sAPPα, of which APP96-110 is the active region, is its high structural similarity to growth-factor like domains, and its strong affinity to heparin, particularly to heparin sulfate proteoglycans (HSPGs) [34–36]. The importance of APP96-110’s affinity for heparin binding for its neuroprotective activity was demonstrated using an APP96-110 analogue with reduced heparin binding affinity, which in turn showed no neuroprotective effect *in vivo* after moderate-severe diffuse TBI [14]. It is thought that APP96-110 could bind to cell-surface or extracellular matrix bound HSPGs to elicit a neuritogeneic response [19, 35, 37]. The APP96-110 region contains a β hairpin loop constrained by a disulphide bond between cysteines 98 and 105 [36, 37], which has been shown to be critical for promoting neurite outgrowth from central and peripheral neurons [37–40], as well as the activation of MAP kinase [41].

Irrespective of the mechanisms of neuroprotection, this study demonstrates that APP96-110 is efficacious by IV administration for up to 5 hours after TBI and strengthens its potential as a novel and multifactorial therapeutic agent following trauma. Important next steps for APP96-110 include enhancing our understanding of its pharmacokinetics to optimize its therapeutic activity, and also identifying its receptor and the signaling pathways it activates, which will help decipher the molecular mechanisms for APP96-110’s activity. Testing the efficacy of APP96-110 in other neuronal injury models such as spinal cord injury and ischemia, where APP expression has been shown to be altered [42, 43], could broaden the therapeutic utility of APP9-110. As such, further development of APP96-110 as a therapeutic for TBI, and in particular DAI that currently lacks an efficacious treatment, is warranted. Overall, APP96-110 shows promise as a novel and clinically relevant treatment option by offering substantial neuroprotective and neurotrophic effects towards reducing secondary injury and functional deficits associated with acute traumatic brain injury.

## Supporting information

S1 File 30 minute rotarod performance. (PZFX)

S2 File 30 minute axonal injury counts. (PZFX)

S3 File GFAP counts. (PZFX)

S4 File Iba1 counts. (PZFX)

S5 File 5 hour rotarod performance. (PZFX)

S6 File 5 hour axonal injury counts. (PZFX)

## Abbreviations

APP: amyloid precursor protein
DAI: diffuse axonal injury
GFAP: glial acidic fibrillary protein
HSPGs: heparin sulphate proteoglycans
IHC: immunohistochemistry
IV: intravenous
TBI: traumatic brain injury
